# Immune Monitoring After Allogeneic Hematopoietic Cell Transplantation: Toward Practical Guidelines and Standardization

**DOI:** 10.3389/fped.2020.00454

**Published:** 2020-08-21

**Authors:** Jaap Jan Boelens, Kinga K. Hosszu, Stefan Nierkens

**Affiliations:** ^1^Stem Cell Transplantation and Cellular Therapies, MSK Kids, Memorial Sloan Kettering Cancer Center, New York, NY, United States; ^2^Princess Máxima Center for Pediatric Oncology and UMC Utrecht, Utrecht, Netherlands

**Keywords:** immune monitoring, immune reconstitution, hematopoietic (Stem) cell transplantation (HCT), cellular therapies, harmonization

## Abstract

Hematopoietic cell transplantation (HCT) is often a last resort, but potentially curative treatment option for children suffering from hematological malignancies and a variety of non-malignant disorders, such as bone marrow failure, inborn metabolic disease or immune deficiencies. Although efficacy and safety of the HCT procedure has increased significantly over the last decades, the majority of the patients still suffer from severe acute toxicity, viral reactivation, acute or chronic graft-versus-host disease (GvHD) and/or, in case of malignant disease, relapses. Factors influencing HCT outcomes are numerous and versatile. For example, there is variation in the selected graft sources, type of infused cell subsets, cell doses, and the protocols used for conditioning, as well as immune suppression and treatment of adverse events. Moreover, recent pharmacokinetic studies show that medications used in the conditioning regimen (e.g., busulphan, fludarabine, anti-thymocyte globulin) should be dosed patient-specific to achieve optimal exposure in every individual patient. Due to this multitude of variables and site-specific policies/preferences, harmonization between HCT centers is still difficult to achieve. Literature shows that adequate immune recovery post-HCT limits both relapse and non-relapse mortality (death due to viral reactivations and GvHD). Monitoring immune parameters post-HCT may facilitate a timely prediction of outcome. The use of standardized assays to measure immune parameters would facilitate a fast comparison between different strategies tested in different centers or between different clinical trials. We here discuss immune cell markers that may contribute to clinical decision making and may be worth to standardize in multicenter collaborations for future trials.

## Introduction

The probability of long-term survival after hematopoietic cell transplantation (HCT) has steadily improved in the last decade with advances in treatments (1, 2). However, long term survival after HCT is still hampered by adverse events, such as graft-versus-host disease (GvHD), infections and relapse of the underlying disease (3–6). Delayed immune reconstitution plays a central role in most of these events (7, 8), suggesting that strategies that increase immune recovery are of great interest to increase survival chances post-HCT (9).

Among the graft-related variables that can influence immune reconstitution are the graft source and composition, degree of HLA-match and the cell dose (10–12). Transplantation with matched related bone marrow (BM) or peripheral blood (PB) (stem) cells is considered the standard for allo-HCT, but also matched unrelated donors (MUD) (mis)matched unrelated cord blood (CB), a haplo-identical or a mismatched family donor can be considered as alternative graft sources. The HCT-source and its manipulation, such as CD34+ positive selection (13) or other *ex vivo* T-cell depletion (14) and *in vivo* T cell depletion with serotherapy [e.g., anti-thymocyte globulin (ATG) or Alemtuzumab] may be important factors defining the probability of T-cell immune reconstitution (IR). Moreover, grafts are selected based on HLA-matching criteria, where the degree of HLA-match is, in general, stricter for BM/PB than for CB grafts, and may depend on the indication. Also, the use of grafts from younger donors is preferred by centers because it associates with better survival chances, due to lower non-relapse mortality (15–17). The graft's cell dose is considered, particularly in CB transplants, because units with low numbers of CD34+ cells were associated with inferior outcomes (18). A retrospective European Society for Blood and Marrow Transplantation (EBMT) analysis by Czerw et al. showed that T cell numbers in the HCT graft are highly variable (range; 50–885 × 10e6/kg) and positively correlate with an increased incidence of grade III-IV acute (a) GvHD (19). Others found that higher numbers of γδT cells (20) in the graft associate with favorable immune reconstitution and superior clinical outcome (21). However, whether the combination of graft source, match grade, cell dose and graft composition will result in optimal immune recovery post-HCT is impossible to deduce from these data. To get more insight into the contribution of different parameters to outcome, we need to identify the best combination of markers that associate with clinical outcome. A reliable predictor for outcomes, across the variety of transplant platforms (e.g., T replete and T deplete), seems to be CD4+ T cell counts above 50/μL within 100 days after transplant (22–24).

A decisive factor that may even overrule the graft-related effects, is the type and timing of the conditioning regimen used. Pharmaco-kinetic and -dynamic studies show strong correlations between post-transplant recovery of immune cells and the timing and dose of conditioning agents [e.g., fludarabine (Flu), busulfan (Bu), and ATG] (25, 26). These data point toward personalized dosing strategies to achieve an optimal exposure in every individual patient (27–29).

Due to the multitude of variables described above and the site-specific policies/preferences, a precise understanding of how these variables can be influenced to optimize outcomes of HCT is difficult to achieve. While donor-selection in cell transplantation has been increasingly standardized over the last decades (30, 31), harmonization of the conditioning regimen is still lacking and the highly variable pharmacokinetics of drugs used in the conditioning regimen and their dramatic effects on outcome are still not widely considered. In the first part of this review, we will provide an overview of the association between different conditioning regimens, outcomes and IR post-HCT. Given the strong relationship between immune recovery and outcome (32, 33), monitoring immune parameters post-HCT may serve as a relatively fast predictor for outcomes (including survival, relapse, and non-relapse mortality) and accelerate comparisons between different strategies in different centers and between different clinical trials. The use of standardized assays across laboratories will be imperative for this purpose. In the second part, we discuss a rationale for selection of a minimal parameter set to monitor immune recovery that could be considered for standardization.

## Conditioning Regimens, Immune Reconstitution, and Outcomes: Toward Individualized Dosing

We recently showed that the pharmacokinetics of rabbit ATG (rATG) is highly dependent on absolute lymphocyte count (ALC) as a representation for the receptor-load (26). In adults, the ALC before rATG-dosing was the only predictor for rATG clearance, while in pediatric patients a weight of <40 kg also influenced clearance. In Table 1 the suggested rATG (Thymoglobulin) exposure targets for pediatric and adult patients are listed. It is important to realize that the ligands recognized by rATG and rATLG (anti-T-lymphocyte globulin; Neovii Biotech, Rapperswil, Switzerland) are not identical, so the target exposures for rATG do not apply for rATLG (26, 35, 36). A nomogram for ATLG should still be made, as its effects are likely similar to those of rATG. Soiffer et al. showed that ATLG was associated with inferior chronic-GvHD-free, leukemia-free survival in a *post-hoc* analysis of a randomized controlled trial with three different regimens (25). This study nicely illustrates that it is essential to understand the effects of all agents on immune reconstitution and the interrelationships between the agents that may significantly alter the outcomes. In this trial, patients receiving total body irradiation (TBI) + cyclophosphamide (Cy) had lower “absolute lymphocyte counts” on the day of rATLG dosing compared to patients receiving a chemo (Bu-Cy or Bu-Flu)-based regimen or patients that received Cy first, before receiving TBI (usually because of practical issues). This resulted in a slower clearance of rATLG, delayed immune reconstitution and subsequently higher transplantation related mortality. Other forms of serotherapy, such as Alemtuzumab, as well as equine-ATG (less frequently used), can have highly variable PK and a significant influence on IR and survival chances.

**Table 1:** Suggested novel ATG (thymoglobulin) dosing nomograms based on PKPD modeling for (non-)myelo-ablative settings in pediatrics and adults [For pediatrics based in PARACHUTE clinical trial; (34)].

**Setting**	**Dosing on**	Target AUC after HCT (AU*d/mL) and donor source	**Starting** **day**	**References**
Pediatrics; myelo-ablative	Weight ALC Cell source	<20 for cord blood <50 for bone marrow (T-Replete)	−9	(26, 34)
Adults: non-myelo-ablative	ALC	60–90 for peripherally mobilized stem cells (T-Replete)	−9	(35)

*Absolute lymphocyte count (ALC). Area under the curve (AUC)*.

A prospective validation of a new dosing nomogram for rATG in pediatric patients (PARACHUTE trial) proved to be effective to expedite immune reconstitution (primary endpoint) and survival (secondary endpoint) (34). This study confirms our previous results showing that a timely recovery of CD4 T cells (>50 CD4+ T cells/μL at two consecutive measurements within 100 days post-HCT) is the strongest predictor of leukemia-free survival, non-relapse mortality, as well as overall survival (23, 35, 37, 38). We previously showed that adequate CD4 recovery is associated with a lower chance for relapse in acute myeloid leukemia (AML) (22) and with higher survival chances for patients with adenoviral reactivation (24). Recently this association was also found to be a significant predictor in a predominantly T cell depleted cohort of pediatric and adult patients (23, 26, 39). Moreover, a recent multicenter trial also showed the association of CD4 T cell recovery with survival chances in patients developing GvHD (23). Interestingly, the CD4 IR is preceded by increasing cell numbers of the myeloid lineage and could even be retraced to the proliferative potential of myeloid cells in BM and CB grafts (38). This makes CD4+ T >50/μL a reliable biomarker for clinical decision making, e.g., patients who fail to reconstitute CD4 T cells may, for instance, be eligible for prophylactic therapy or for pre-emptive treatment upon the first positive virus measurement. Alternatively, patients with adequate CD4 T cell numbers should be monitored carefully, but would not receive treatment, as there is a reasonable chance that the reconstituting immune system will clear the pathogen by itself. Of course, these observations should be further studied in the context of a clinical trial, and follow-up studies and validations are needed to confirm whether predictions can be further improved.

In a recent retrospective cohort analysis (including > 650 pediatric and young adult patients), the cumulative exposure to Bu significantly influenced clinical outcomes (40). The optimal Bu-exposure (80–100 mg^*^h/L) was associated with the highest survival chances and lowest toxicity and was independent of the indication, chemo-combination (Bu + Flu, Cy + Bu, or Bu + Cy and Melphalan), age and donor source. The method of Bu-exposure estimation may differ between centers and could be responsible for a large variation in the reported estimated exposures (40). This emphasizes the value of standardizing the entire process of sample logistics up to data reporting, in order to be able to compare different treatment strategies.

More recently, Flu-exposure (given prior to transplant) was also found to influence survival by negatively affecting IR in patients who were over-exposed (41, 42). This is an interesting observation, given that the exposure in blood was only before transplant, and it is important to note that the PK in the organs in experimental models can be different, as discussed by the authors. These studies show that the pharmacokinetic variation between individuals is high and that these differences in exposure can have significant impact on outcomes, including survival. It is still daily practice that a variety of conditioning regimens are used, which can complicate comparisons of HCT outcomes across different centers and even within trials as illustrated by Soiffer et al. (25). Also, post-transplant Cy is a frequently used (and a simple and cheap) transplant platform to cross the HLA barrier in allogeneic HCT and induce a state of immunologic tolerance (43, 44). While its simplicity makes it an attractive approach, there is not much data available about immune reconstitution in this transplant setting, which would be of interest to study in more detail to identify predictors for failure or success.

Together, these data suggest that the recovery of (CD4+) T cells may be an easy-to-obtain biological marker and a potential biomarker/predictor to monitor treatment success in population studies. Additional work is needed to identify further biomarkers with clinical predictive value. A biological predictor for clinical outcomes, even before clinical signs will be visible, may be valuable to anticipate graft-versus host responses. It may also facilitate stratification of patient subgroups for treatment interventions after HCT, e.g., prophylactic antiviral therapy, or the use of checkpoint inhibition, to ensure adequate treatment for responsive patients, while predicting non-responders or patients with a high probability of developing life-threatening side effects who may be directly eligible for other treatments (45). As different institutions have their own policies on sample collection and monitoring, it is of crucial importance to set up multicenter validation trials to establish standardized protocols for sampling, handling logistics, measurements and data processing, to reduce result variability and allow for more accurate data comparison.

## Monitoring Immune Cell Reconstitution

Multicolor flow cytometry is the technology by default in many accredited transplant laboratories for immune cell reconstitution monitoring. It enables the analysis of a large number of parameters simultaneously, in a short time and for a reasonable cost. Many centers monitor the recovery of the common leukocyte subsets based on CD45 (leukocytes), CD3, CD4, CD8 (T cells), CD19 (B cells), αβTCR, γδTCR and CD16/CD56 (NK cells), and may analyze the maturation of T cells from naïve to effector/memory cells to discriminate lymphopenic proliferation and the recovery of thymic output. There are, however, no stringent guidelines for clinical decisions based on quantification of cell subsets post-HCT.

Many effector and regulatory cell types have been linked to the anti-cancer immune responses (46). Different subsets of T cells play crucial roles in controlling disease progression, and effectors like CD8+ and CD4+ T cells have been associated with direct anti-tumor activity and a favorable prognosis, particularly when those T cells express memory and activation markers (47–50). NK cells were shown to mediate tumor regression in AML patients, eliminate graft rejection and protect patients against GvHD (51, 52). The presence of mature antigen-presenting dendritic cells (DCs) has been correlated with improved survival (53–56). T regulatory (Treg) cells down-modulate T-cell activation through the production of immunosuppressive cytokines (TGFβ, IL-10), as well as through surface receptors (CTLA4), and can drastically impact both the anti-tumor immune response as well as the control of GvHD (57–59). Myeloid suppressive cells (60–62) and regulatory B cells (63–65) also play a potential role in attenuating the immune response and controlling effector cells and signaling mechanisms. Moreover, the presence of certain biomarkers may be predictive for the functioning of effector mechanisms; e.g., sorafenib-related IL-15 production causes an increase in CD8+CD107a+IFN-γ+ T cells with features of longevity and eliminates leukemia in secondary recipients, indicating that sorafenib after HCT might be more effective through induction of IL-15-mediated metabolic reprogramming of leukemia-reactive T cells (66). Others showed that the presence of peripheral blood DCs in high frequencies relates to clinical response to high-dose IL-2 (67). This data suggests that DCs may be instrumental for endogenous- and immunotherapy-induced immunity against cancer.

In summary, data from multiple studies suggest that monitoring immune cell subsets may hold predictive value for outcome, development of adverse effects, or may be used for patient stratification for certain treatment modalities. Multicenter validation studies are required using standardized protocols for sampling, handling logistics, measurements and data processing. Diagnostic laboratories in transplant centers have standardized protocols to diagnose and measure minimal residual disease burden in patients with hematological malignancies. Most centers provide validated assays for CD34 cells and immune monitoring of leukocyte subsets post-HCT in a standardized way. It thus seems a small step to collect and collectively report these data in international databases (e.g., EBMT), and to relate biological markers, such as CD4 T cell reconstitution, to outcome parameters and to the treatment procedures.

## Cytokine Profiling

Profiling soluble markers in blood may be of value to assess the status of the immune system before, during and after immunotherapeutic interventions for infections or GvHD, or to provide additional insight into the therapeutic mechanisms of action. Blood markers should be regarded as surrogate markers and may not always reflect local responses in affected tissues, such as skin or gut in GvHD. As this topic has been reviewed recently (9, 68), we here just describe two scores that may be close to multicenter validation studies and emphasize the standardization of techniques. Previous studies characterized biomarkers for aGvHD-related mortality post-HCT; a biomarker score using ST2, TNFR1, and Reg3a to guide risk-adapted therapy at aGvHD onset irrespective of the conditioning regimen intensity (69, 70). Another formula, including the markers lactate dehydrogenase, creatinine and thrombocytes, termed the Endothelial Activation and Stress Index (EASIX), was also reported useful for prognosis of survival chances in patients suffering from aGvHD after HCT with reduced intensity conditioning (71). These scores should still be prospectively validated in the different HCT settings, preferentially in coordinated multicenter trials before they can be implemented in patient care.

The technology to acquire the parameters for EASIX may be more standardized in and between different centers than those for analyzing proteins, such as ST-2. Many technologies are available e.g., antibody-based ELISA's or multiplex platforms, liquid chromatography—mass spectrometry (LC-MS), electro-chemiluminescence (72), but concentrations may differ depending on the methods used. The latter is problematic for implementation of mathematical scores. Standardization is needed for the procedures of sample processing and isolation, selection of tubes and duration of storage/cryopreservation. Protein levels can differ considerably between serum and plasma samples (due to release of platelet-associated molecules into serum), even between the type of anticoagulant used, and are prone to changes due to variations in time (from sampling to processing and time of storage) and/or temperature (73).

## In Summary

Survival after HCT depends on many factors, including a balanced and timely immune reconstitution in the first 3 months post-HCT. Delayed immune reconstitution is associated with a lack of disease control, viral infections, and seems to increase the chances of immune dysregulation, resulting in higher non-relapse mortality rates. Currently, only a few HCT programs consider therapeutic drug monitoring of agents used in the conditioning (a main factor influencing immune recovery) or apply frequent systematic monitoring of immune cell reconstitution to predict unwanted events.

Based on current knowledge, recommendations can be made with regards to personalized drug dosing and application of a standardized minimal panel for immune monitoring to generate fast responsive surrogate markers for efficacy or the development of unwanted effects. It may be valuable to register the details of the treatment modality, i.e., drug doses and if possible, drug exposure, graft composition, and standardized immune reconstitution parameters to the Center for International Blood and Marrow Transplant Research (CIBMTR) and EBMT databases. This could also initiate the establishment of consensus guidelines in clinical trials on monitoring and reporting a minimal set of parameters, which can be extended to add-on trial-specific parameters (Table 2). Efforts to harmonize HCT protocols/platforms aiming to create the optimal “immune milieu” to exert the most optimal effector mechanism may have more impact on survival chances than many novel maintenance therapies (35, 74). Moreover, it will provide a more straightforward comparison between different treatment modalities, due to better prediction of the immune milieu.

**Table 2:** Suggested harmonized panels.

	**Cell type**	**Standard panels**	**Advanced monitoring** **suggestions**
Cell Phenotyping of Graft Composition and IR	αβT γδT Treg B NK/NKT DC/mono	αβTCR, CD45RO/RA, CD3, CD4, CD8, CD27 γδTCR, CD45RO/RA, CD3, CD27 CD45, CD3, CD4, CD25, CD127, FoxP3 CD45, CD19, CD38, CD27, IgM/G/D, CD21 CD45, CD3, CD56, CD16 CD11c, HLA-DR, CD14, CD16, CD1c, CD141, CD303	•Intracellular cytokines after PMA/ionomycin stimulation •Specific TCR by multimer approach. •TCRγδ
Secretome	-		MultiPlex panel (e.g., IL-7, IL-15, ST2, TNF-a, IL-6, HGF, IL-2R, IL-8, GM-CSF, etc.)
Cell function	-		•NK cell lysis •T cell proliferation after stimulation with antigens and mitogens •B cell maturation

*General parameters that could be included in harmonized immune monitoring protocols across most studies/centers, and advanced parameters that may be of great value in specific studies that can only be performed in specialized immunology labs or analyzed in a central laboratory*.

## Author Contributions

All authors listed have made a substantial, direct and intellectual contribution to the work, and approved it for publication.

## Conflict of Interest

JB has received consulting fees from Race Oncology, Takeda, Omeros, Advances Clinical, Bluebird Bio, Bluerock, Sanofi (all not related to research described in this review). The remaining authors declare that the research was conducted in the absence of any commercial or financial relationships that could be construed as a potential conflict of interest.
